# The Effect of Saffron Kozanis (*Crocus sativus* L.) Supplementation on Weight Management, Glycemic Markers and Lipid Profile in Adolescents with Obesity: A Double-Blinded Randomized Placebo-Controlled Trial

**DOI:** 10.3390/children10111814

**Published:** 2023-11-15

**Authors:** Eleni P. Kotanidou, Vasiliki Rengina Tsinopoulou, Styliani Giza, Stergianna Ntouma, Chrysanthi Angeli, Michail Chatziandreou, Konstantinos Tsopelas, Ioulia Tseti, Assimina Galli-Tsinopoulou

**Affiliations:** 1 2nd Department of Pediatrics, School of Medicine, Faculty of Health Sciences, Aristotle University of Thessaloniki, AHEPA University General Hospital, Stilponos Kyriakidi 1, 54636 Thessaloniki, Greece; 2 Uni-Pharma S.A., 14564 Kifissia, Greece

**Keywords:** crocus, saffron, adolescent, youth, obesity, obese, prediabetes, glucose, BMI, insulin

## Abstract

Global rates of adolescent obesity have led the World Health Organization to consider the disease a pandemic that needs focus. In search of new anti-obesity agents, Crocus sativus, popularly known as saffron, is a nutraceutical agent, praised for its beneficial effects. The study aimed to investigate the possible effect of Kozanis saffron administration on weight management of obese prediabetic adolescents. Seventy-four obese prediabetic adolescents participated in a double-blind placebo-controlled trial of three arms, randomly assigned to receive either Kozanis saffron (*n* = 25, 60 mg/day), metformin (*n* = 25, 1000 mg/day) or a placebo (*n* = 24), for twelve weeks. Anthropometry, glycemic markers and lipid profiles were investigated at baseline and post-intervention. Saffron supplementation significantly reduced the weight z-score, BMI, BMI z-score and waist circumference (WC) of obese adolescents; however, this reduction was less significant compared to the effect of metformin. Metformin administration offered a significantly more profound improvement in anthropometry compared to saffron administration. Saffron administration also provided significant improvements in weight, weight z-scores, BMI values, BMI z-scores and WCs compared to the placebo. Saffron supplementation failed to change any glycemic marker, but provided a significant reduction in fasting triglyceride levels and also a significant increase in fasting HDL levels. Saffron Kozanis constitutes a promising nutraceutical option for adolescents and children with obesity and prediabetes in need of weight management.

## 1. Introduction

Obesity constitutes one of the most prevalent public health issues among all populations and age groups worldwide, resulting in significant increases in mortality and morbidity associated with cardiovascular disease, diabetes, stroke, and cancer [1,2,3,4]. The World Health Organization (WHO) prioritizes our actions against obesity in children and adolescents rather than in adults, setting distinct targets to be met [5]. Adolescence, the transitional period that bridges childhood and adulthood, represents a crucial time frame that deserves to be exploited as an opportunity to promote and establish both health and an optimal lifestyle. Both lifestyle modification and pharmacotherapy are considered by health care professionals as treatment options for weight management. The complex and unsolved pathophysiological roots of obesity force the need to develop interventions with high rates of long-term compliance.

Herbal dietary supplements are among the most common complementary medicine methods recommended for weight management [6]. Medicinal plants are agents that present a very popular perception by a wide range of people. This popularity is linked to the speculation that “nature is both wise and safe”. In the era of evidence-based medicine, a focus on examining this hypothesis with statistical and experimental tools is of major importance, with the aim of enriching our therapeutic options. Additionally, pharmaceutical agents for weight management in children and adolescents are very few and strict age restrictions usually apply. On the other hand, the disappointing data on weight regain after the discontinuation of pharmacotherapy compel the research community to pursue new, safe, and outstanding therapeutic alternatives to combat the obesity epidemic in childhood and adolescence. Medicinal plants have gained scientific interest in the last decade, as a potentially effective alternative for the management of excess body weight.

Crocus, the dried stigma of *Crocus sativus* L. from the Iridaceae family, is recognized as a powerful medicinal plant. The plant is an ancient spice, cultivated in specific areas of the world during the last 3000 years. The name of the flower originates from the Greek mythical god Hermes and his friend Crocus who was fatally injured in a discus throwing game and the droops of his blood gave birth to the flower [7]. The cultivation of the plant is a notably cost-intensive process, since 175 flowers are needed to obtain one gram of saffron [7]. It is cultivated in a few countries included Greece (prefecture of Kozani), producing a high-quality crop of the plant, named Crocus Kozanis [8]. The plant contains more than 150 volatile and non-volatile compounds [9]. The main volatile compounds are terpenes, terpenic alcohols and their esters [9]. The non-volatile chemical components of stigmas of *Crocus sativus* L. flower contain the following three main metabolites: (1) *Crocins* (8′-diapocarotene-8,8′-dioic acid), the saffron-colored compounds; (2) *Picrocrocins* (C_16_H_26_O_7_), the main factor accounting for saffron’s bitter taste; and (3) *Safranal* (2,6,6-trimethylcyclohexane-1,3-dien-1-carboxaldehyde), responsible for saffron’s aroma [9]. Crocin and picrocrocin are the main compounds in saffron. Crocin is responsible for its characteristic color, and picrocrocin is a precursor of safranal. In addition to these, carotenoids (lycopene, alpha-, beta-, gamma-carotene, zeaxanthin, phytoene, phytofluene, mangicrocin and xanthone-carotenoid glycosidic conjugate), phenolic anthocyanins, flavonoids, vitamins (riboflavin and thiamine), amino acids, proteins, starch, mineral matter, gums, and other chemical compounds have been found in saffron [9,10].

Existing literature suggests saffron as a promising therapeutic agent in clinical trials of adult populations with either obesity alone or obesity with dyslipidemia, prediabetes or diabetes mellitus type 2 [11,12,13,14,15,16,17,18,19,20]. High-quality evidence has recently emerged through meta-analytic approaches, providing data that saffron supplementation in obese adults can significantly modify their anthropometry by reducing their waist-to-hip ratio (WHR) [21]. Weight loss and improvement in BMI after saffron supplementation are also frequent but not constant findings in adult obesity trials [21]. Improvement of the lipidemic profile is another proven effect following saffron administration in adults with obesity, having a documented reduction in serum cholesterol and triglyceride concentrations, alongside an increase in serum HDL levels [22].

To our knowledge, no clinical trial has so far investigated the effect of saffron on adolescent obesity or adolescent dysglycemia, either in terms of anthropometry or biochemistry, as expressed by serum lipid profiles and serum glycemic indices. This randomized, placebo-controlled, double-blind clinical trial aimed to investigate the effect of Saffron Kozanis oral solution on body weight management and serum glucose and lipid concentrations in adolescents with obesity and prediabetic states after 12 weeks of intervention.

## 2. Materials and Methods

### 2.1. Study Design

In this randomized double-blind, placebo-controlled clinical trial (allocation ratio 1:1:1), obese prediabetic adolescents were recruited voluntarily from the Unit of Pediatric Endocrinology and Metabolism of the 2nd Department of Pediatrics, School of Medicine, Faculty of Health Sciences, Aristotle University of Thessaloniki, AHEPA University General Hospital, Thessaloniki, Greece. The recruitment period was from May 2021 to January 2023. Written informed consent was obtained from all parents and guardians, based on the adolescents’ consent and willingness to participate in the study.

The study was conducted in accordance with the Declaration of Helsinki and subsequent revisions [23] and was approved by the institutional review board. This research project was approved by the Ethics Committee of the School of Medicine, Faculty of Health Sciences, Aristotle University of Thessaloniki (code 6230-29-07-20). The study was also registered in the Registry of Clinical Trials of the US National Library of Medicine (trial ID: NCT05572749).

### 2.2. Inclusion Criteria, Exclusion Criteria and Definitions

Criteria for the diagnosis of obesity were based on the current Endocrine Society clinical practice guideline, endorsed by the European Society of Endocrinology, that defines obesity as a BMI ≥ 95th percentile in the Centers for Disease Control and Prevention normative percentiles [24]. Criteria for screening and diagnosis of prediabetes were according to the American Diabetes Association’s current Adolescent Diabetes Standards of Care guideline, based on the presence of impaired fasting glucose and/or impaired glucose tolerance and/or HbA1c levels between 5.7 and 6.4% [25]. Standards of care American Diabetes Association guidelines were applied to define IFG (glucose fasting levels from 100 to 125 mg/dL) and IGT (2 h post load during an oral glucose tolerance test glucose level from 140 to 199 mg/dL) [25]. Inclusion criteria were as follows: adolescents of Caucasian origin, aged 10–18 years with obesity according to aforementioned criteria, who were screened for the first-time for prediabetes and fulfilled the above-mentioned diagnostic criteria for prediabetes. Exclusion criteria were defined as follows: non-Caucasian origin, previously diagnosed prediabetes, past exposure to any anti-diabetic medication, presence of any chronic disease other than obesity, administration of any chronic medication during the last year before recruitment, use of any dietary supplement during the last year before recruitment, previous use of saffron in the diet with a frequency of more than once per 90 days in the last year before recruitment and a known allergy to saffron.

### 2.3. Sample Size Calculation

Estimation of the appropriate sample size was performed using power analysis software (ClinCalc.com, available at https://clincalc.com/stats/samplesize.aspx, accessed on 1 May 2021). Rationale was based on previously published data that observed significant changes in BMI values following saffron supplementation in obese adults (μ1 ± SD = 29.46 ± 70.84, μ2 ± SD = 30.81 ± 0.91), since the present study is the first to report the effect of saffron administration in obese adolescents using BMI values [11,21]. The analysis revealed that a sample size of at least 12 participants per group (total population, *n* = 36) was needed to provide power (1 − β) of 0.80 (α = 0.05). This number was increased to 25 samples per group to account for expected dropouts.

### 2.4. Anthropometric and Clinical Assessment

After overnight fasting, obese adolescents underwent a standard oral glucose tolerance test and blood samples were obtained every 30 min for 2 h after glucose load, in order to screen for prediabetic state according to the ADA guideline [25]. Individuals who met the diagnostic criteria of prediabetes were further screened for study inclusion and exclusion criteria, and invited to participate in this trial. Adolescents, their guardians and parents and the assessor researchers were all blinded to the allocation during the study.

Adolescents were randomized to receive saffron Kozanis, metformin or a placebo in a 1:1:1 ratio using a computer-generated random number code. The randomization and allocation process were performed by the trial’s principal investigators (E.P.K, A.G.T.) who were not involved in the material allocation or measurement process. Assignments were kept in sealed, opaque envelopes in each participant’s hard-copy file, until the point of the data analysis. All participants were offered a 20 min consultation with the same clinical nutritionist on the day of randomization as an educational tool in healthy eating behaviors.

For twelve weeks, participants in the three study groups were instructed to take their randomly assigned intervention: Saffron Kozanis oral solution, metformin oral solution or placebo oral solution. After the allocation process, study intervention materials were offered for 12 weeks to the adolescent participants (S.G., S.Ν., C.A., M.C., K.T.). Subjects allocated to the Saffron Kozanis intervention group were instructed to follow a dosage regimen of 5 mL of saffron oral solution twice per day, corresponding to 30 mg of saffron twice daily. Subjects allocated to the metformin intervention group were instructed to follow a dosage regimen of 200 mL metformin oral solution after preparation of an effervescent tablet once daily, corresponding to 1000 mg of metformin once per day. Subjects allocated to the placebo group were instructed to follow a dosage regimen of 5 mL of placebo oral solution twice per day. The daily dose of saffron was considered based on previous studies investigating the effect of saffron sativus in the pediatric population using a clinical trial design [26,27] The twice-daily administration regimen was considered based on the vast majority of previous trials investigating the effect of saffron in adult populations with obesity and diabetes [21]. According to the US Food and Drug Administration, saffron has been considered a safe substance since 2012, whereas daily doses of up to 1.5 g are safe according to the WHO [9].

Weight and height were measured by a blinded assessor (E.P.K., V.R.T.) (SECA 711 scale, Hamburg, Germany; Harpenden stadiometer, Veeder-Root, Elizabethtown, NC, USA) and BMI was calculated. To ensure reliability of anthropometric measurements, all participants were measured with the use of the same scale and anthropometer, from the blinded assessors. Weight and height z-scores for age and gender were quantified at baseline and the end of the study for all participants [28]. The estimation of the degree of obesity was quantified after calculating the BMI z-score value of the subjects for age and gender [28]. The Center for Disease Control and Prevention growth charts served as reference data for all z-score quantifications. Waist circumference was measured to the nearest 1 mm with an anthropometric non-elastic tape measure, after normal expiration, at the level of the umbilicus (WHO method). Participants underwent a complete physical examination. Blood pressure was measured three times, with 1 min intervals, from the non-dominant arm in a seated and relaxed position after 5 min of rest, with a digital sphygmomanometer (DINAMAP, Johnson & Johnson, Medical INC, Arlington, TX, USA). To ensure reliability of a blood pressure measurements, the same sphygmomanometer was used in all participants, whereas the mean of three distinct measurements was recorded as blood pressure.

### 2.5. Biochemical Measurement

After overnight fasting, a blood sample was collected from all participants at baseline and the end of the intervention. Fasting blood glucose was analyzed immediately at the bedside by an automatic analyzer (GlucoMen Areo 2k, Menarini Diagnostics s.r.l, Florence, Italy) using fresh blood. Serum biochemical parameters were assessed using standard methods and an ARCHITECTc 16,000 clinical chemistry system (Abbott, Abbott Park, IL, USA). Fasting lipid profiles (total cholesterol, high-density lipoprotein (HDL) cholesterol, low-density lipoprotein (LDL) cholesterol and triglycerides), urea, uric acid and creatinine levels were measured. Fasting insulin concentrations were measured with an ADVIA Centaur XPT Immunoassay System (Siemens Healthcare GmbH, Erlangen, Germany). HbA1c levels were measured using the HPLC method in a Menarini ARKRAY ADAMS™ A1C HA-8180 Analyzer (Menarini Diagnostics s.r.l, Florence, Italy). The homeostasis model assessment index for insulin resistance (HOMA-IR) was calculated with the following formula: [fasting glucose in mmol L – 1 × fasting insulin in mIU/L/22.5] [29].

### 2.6. Crocus sativus L. Preparation

In the present study, two different liquid preparations were prepared (I.T.) on a large scale by the Uni-Pharma Kleon Tsetis Pharmaceutical Laboratories S.A. member of the Tsetis Group of Pharmaceutical Companies (OFET), including an oral suspension of saffron and the corresponding placebo. As the active ingredient, saffron stigma powder was used from Kozani Saffron Producers Cooperative (Cooperative de Saffran, Kozani, Greece). Both preparations consisted of two solvents, a preservative (0.3% *w*/*w*), a viscosity increasing/suspending agent, flavor enhancers and colorants. Saffron stigmas were added to the first formulation and not to the placebo preparation, while colorants were added to the placebo only. The batch size of both formulations was 50 L, which were then filled into 150 mL amber glass bottles.

The production process followed consisted of simple consecutive steps. In more detail, a 100 l stainless steel tank of the first solvent was added and left under stirring at 200 rpm for 5 min at ambient temperature. The flavor enhancers and the preservative were then added and stirred until completely dissolved. The next step was to add the second solvent and the suspending agent, which was left under stirring until complete dissolution, for approximately 4 h. Saffron was then added to the solution and stirred until a homogenized suspension was visible. On the contrary, in the placebo, instead of saffron, coloring agents were added to simulate the orange-red color of the saffron suspension. The final step of the procedure in both liquid preparations was to adjust the pH of the solution to 4.5 and to bring the final volume to 50 l by adding the remaining solvent. The final preparations were filled into the glass bottles by an automated bottling, capping and labelling machine. The suspension filled in each bottle was 120 mL.

The appearance of the placebo oral solution (including color, bundling, odor) was similar to the Saffron Kozanis supplement.

### 2.7. Statistical Analysis

Statistical analyses were performed using SPSS software version v24.0.0.2 (SPSS Inc., Chicago, IL, USA). The Shapiro–Wilk test was applied to examine the normality of distribution of continuous quantitative variables. Data for continuous quantitative variables are expressed as mean ± standard deviation. Data for dichotomous variables are expressed as absolute numbers (percentage). One-way analysis of variance (ANOVA) or its non-parametric analogue, the Kruskal–Wallis test, were applied to examine for group differences in continuous data at baseline. The chi-square test was used to examine differences in the frequency of dichotomous data at baseline.

Post-intervention differences across the study groups were examined using ANCOVA models after adjustment for confounding factors age and gender. The paired sample *t*-tests, or its non-parametric analogue Wilcoxon signed-rank test, were used to compare quantitative variables pre- and post-intervention in each group. Differences in pre- and post-intervention variables were calculated for each participant using the following formula: (post-intervention value -baseline value). Comparison of differences was performed by one-way ANOVA or the Kruskal–Wallis test when comparing three groups, or by Student’s *t*-test or Mann–Whitney U test when comparing two groups. *p*-values less than 0.05 were reported as statistically significant.

## 3. Results

### 3.1. Baseline Characteristics of Participants and Intervention

**Figure 1:**
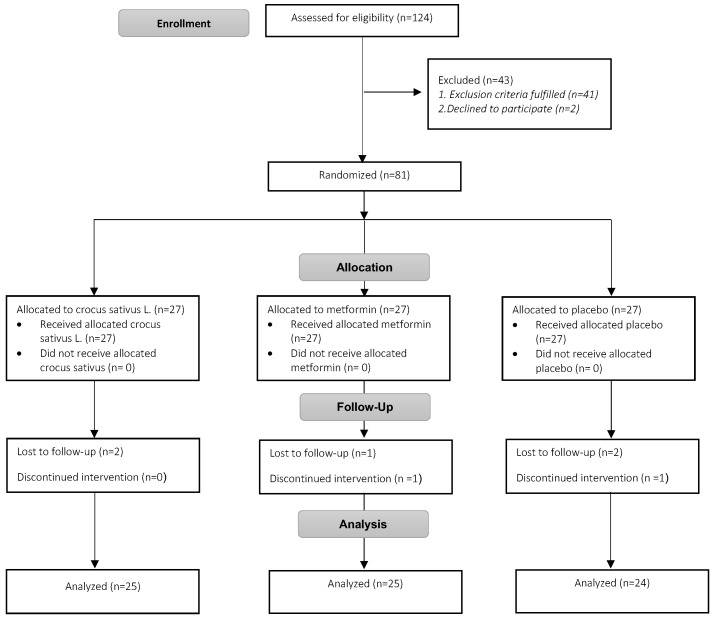
Study flowchart.

Of 124 screened adolescents, 81 were eligible to participate in the study and were randomly assigned to receive saffron, metformin, or the placebo. In total, 74 adolescents (25 in Crocus sativus group, 25 in metformin group and 24 in placebo group) completed the study protocol, as shown in detail in Figure 1, while 7 adolescents withdrew from the study (2 in the Crocus sativus group, 2 in the metformin group and 3 in the placebo group) due to inability to attend the follow-up, withdrawal or unwillingness to continue (Figure 1).

No adverse events or adverse symptoms were reported by the subjects or their guardians in any study group during the study. Baseline general and demographic characteristics of the adolescent participants are summarized in Table 1. No significant difference was observed in the demographic data among the groups, or in the clinical or biochemical profiles at baseline of the study.

### 3.2. Demographic and Anthromopetricindices of Participants before and after Intervention

Demographic and anthropometric indices at baseline and at the end of the study are summarized in Table 2. Mean age, body weight, height, body mass index (BMI), BMI z-score and waist circumference did not differ among the study groups at baseline. Blood pressure values were also similar among participants at the beginning of the study. After three months of intervention, no significant difference was observed in the demographic data or in the anthropometric profiles among the three groups of the study (Table 2). During the intervention period, there was also no significant change in the anthropometric measures after adjustment for age and gender in the three intervention groups (metformin, saffron and placebo) (*p*-value > 0.05), except for a statistical trend to lower the mean BMI z-score in the saffron and metformin groups, compared to the placebo (*p*-value = 0.052).

Within group comparisons revealed that metformin and saffron supplementation significantly reduced the weight z-score, BMI, BMI z-score and WC (*p*-values < 0.05) of the participants, while no significant change in the placebo group occurred (Table 2). The metformin group showed reduced weight (*p*-value < 0.001) and DBP (*p*-value = 0.037), whereas no change was observed in the saffron or placebo groups.

After three months of intervention, the mean post-intervention difference of anthropometric indices (weight, weight z-score, BMI, BMI z-score) was significantly higher in the metformin group compared to the saffron group (Table 3). Only WC presented a similar mean difference post-intervention between the saffron and metformin group. However, it is noteworthy that, when comparing the saffron to the placebo group, the mean difference of all anthropometric values (weight, weight z-score, BMI, BMI z-score, WC) was also higher in the saffron group compared to the placebo group (Table 3). Blood pressure did not present any difference post-intervention in any of the three groups.

### 3.3. Glycemic and Lipidemic Profile before and after Intervention

At baseline, the glycemic and lipidemic profiles did not differ among the three groups (Table 4). Between-group analysis showed no difference among the metformin, saffron or placebo groups in any of the laboratory parameters measured at the end of the study, after adjustment for age and gender. Fasting insulin levels and the homeostasis model assessment for insulin resistance index (HOMA-IR) values presented only a statistical tendency to differ among the three groups when comparing pre- and post-intervention time points (Table 4).

Within group comparisons revealed that the metformin group was the only one to significantly reduce fasting glucose, fasting insulin, fasting cholesterol, LDL cholesterol and HOMA-IR levels at the end of the intervention compared to the baseline (Table 4). The saffron group failed to change any of the laboratory parameters measured, after the intervention, expect fasting triglycerides, which were significantly changed post-intervention compared to baseline. Similarly, HDL was also significantly higher post-intervention compared to pre-intervention in the saffron group. It is noteworthy that even the placebo group managed to present a trend of weight reduction before and after the intervention, obviously due to the placebo effect (Table 4).

The mean difference of all the laboratory investigations was apparently higher in the metformin group when comparing baseline and post-intervention time points (Table 5). The pre- and post-intervention difference was similar between saffron and placebo in all values, except from the fasting insulin levels, HDL and triglyceride levels, which were found to be significantly more reduced after saffron intervention compared to the placebo group (Table 5). In parallel, fasting cholesterol and fasting LDL differences pre- and post- intervention were found to be significantly higher in the metformin group compared to the saffron group. Urea, Uric acid and creatinine levels presented no difference in any of the groups, at any time point of the intervention.

## 4. Discussion

The present study is the first report on the effect of saffron on body weight in the non-adult population of adolescents with obesity. The study aimed to investigate the effect of saffron administration in comparison to the effect of metformin or placebo administration. After three months of intervention, saffron supplementation significantly reduces the weight z-score, BMI, BMI z-score and WC of obese adolescents; however, this reduction is less significant compared to the effect of metformin on weight management. Metformin administration provided a significantly more profound amelioration of anthropometry in terms of weight, weight z-score, BMI and BMI z-score compared to saffron administration. However, saffron administration also provided significant improvements in anthropometric values such as weight, weight z-score, BMI, BMI z-score and WC compared to placebo administration. Saffron supplementation fails to alter any glycemic index, but provides a significant reduction in fasting triglycerides and also a significant increase in fasting HDL levels.

Obesity ranks among the non-communicable diseases with high rates worldwide, reflecting the consequences of an unbalanced energy equilibrium [30]. Previously, the disease was contexed in the ultra-simple rational of the excessive fat accumulation as the result of an imbalanced equilibrium between energy expenditures and energy load. Recent evidence supports an ultra-complex rational in which the crucial player for both onset and progression of the disease relates to the gut microbiome [31]. A growing body of evidence, in animal and human protocols, sheds light on the pivotal role of the gut microbiome in glucose and insulin metabolism dysregulation in the context of obesity [31,32]. Thus, gut dysbiosis is proposed as a key factor for the development of chronic non-communicable diseases like diabetes and metabolic syndrome [32,33].

There are only a few approved anti-obesity drugs; thus, an urgent need for new effective anti-obesity agents is evident [34]. Apart from pharmacology, research on non-communicable diseases focuses on the potential effects of functional foods [30]. The aim is to study how their bioactive compounds called nutraceuticals can benefit the population in the prevention or treatment of non-communicable diseases from a young age when incorporated into their diet. The popularity of nutraceuticals among patients has led to a wide range of available research data, which have recently been meta-analyzed in comparative network analyses, in order to form a solid evidence base [35]. Saffron has been discussed as a nutraceutical product with potential anti-obesity activity in the last decade [10]. Its anti-obesity properties are linked to evidence mainly from basic research in four different pathophysiological pathways [10,34]. The proposed anti-obesity mechanisms are based on the fact that saffron reduces food and calorie intake by minimizing dietary fat through the inhibition of pancreatic lipase [10]. On the other side, saffron also presents an anorexigenic effect and a reduction in snacking behavior, through the upregulation of neurotransmitters involved in feelings of satiety and fullness [34]. Additionally, saffron exhibits a weight-reducing effect by suppressing adipocyte differentiation as a very powerful anti-oxidant [10]. Finally, the effect of saffron on weight loss is attributed to the enhancement of glucose and lipid metabolism [34]. Therefore, it is speculated that saffron as well as its active ingredients have a broad spectrum of therapeutic potential, which should be investigated in clinical settings.

The value of saffron in human weight management has been extensively studied, with various protocols in obese adults outlining weight reduction following dietary supplementation [21,36,37]. Meta-analyses of all the available anthropometric data have shown that saffron administration can reduce body weight in adults, but this reduction was only substantially significant in one meta-analytic model [36], while two other meta-analyses also report weight loss that did not reach significance [21,37]. In the same context, the decrease in adult BMI in the pooled data on adult obesity was also evident but not statistically significant [36]. However, more specific anthropometric markers of obesity like waist circumference and weight-to-hip ratio (WHR) were also reported to be significantly improved in obese adults after saffron intake. The most recent meta-analysis documented that WHR was significantly reduced at the 40% level, showing no heterogeneity in the pooled data [21]. Similarly, saffron supplementation was also capable to significantly reduce waist circumference in meta-analytical studies in adults [36]. The present study explores for the first time the effect of saffron on the weight management of obese adolescents and provides the first evidence of a modest but significant reduction in the weight z-score, BMI z-score and waist circumference in obese adolescents, paralleling the findings in adults.

The glycemic profile of obese adults following saffron intake has also been the focus of basic and clinical nutraceutical research. In the present protocol, no benefit was documented for adolescents with obesity and prediabetes after three months of saffron supplementation. Adult clinical data, pooled in meta-analyses, report different findings. In four different meta-analyses published from 2020 to 2022, the administration of saffron/crocin in adults presents the beneficial effect of lowering fasting blood glucose levels significantly, compared to the placebo, although high heterogeneity was evident in the aforementioned studies [21,38,39,40]. The source of heterogeneity among previous meta-analyses is multiple, since the pooled data originated from different clinical conditions: adults with overweight, obesity per se, metabolic syndrome, coronary artery disease, prediabetes, type 2 diabetes and others [21,38,39,40]. The extrapolation of this finding is questionable since the pathophysiology of the different clinical settings is distinct. It is also important to highlight that there are two meta-analyses published in 2019 that report a non-significant change in the glycemic profile of adults after saffron intake, also including data from a wide range of different clinical conditions [22,36]. Likewise, evidence regarding the effect of saffron on glycated hemoglobin (HbA1c%) is conflicting. Three systematic reviews in adult settings concluded non-significant changes in HbA1c% after saffron supplementation [21,36,39]. However, there is a systematic review published by Sohaei et al. reporting a significant reduction in HbA1c% with significant heterogeneity observed among trials [38]. Given the above, a precise conclusion cannot be reached through the currently available data regarding the glycemic status of adults after saffron intake.

Changes in the lipid profile following saffron administration in adults with obesity or diabetes have also been extensively studied. Among the five available meta-analyses evaluating the effect of saffron on total cholesterol levels in adults [21,22,36,40,41], three models reach the conclusion that saffron supplementation can significantly and substantially reduce total cholesterol levels in overweight or obese adults with coronary artery disease [21], as well as lowering total cholesterol levels in mixed adults with either obesity, diabetes, metabolic syndrome, coronary artery disease or schizophrenia [22,40]. The present study in obese prediabetic adolescents failed to demonstrate a significant reduction in total cholesterol levels. However, after saffron supplementation, the adolescents in this study presented a significant reduction in fasting triglyceride levels and also a significant increase in fasting HDL levels.

Five systematic reviews [21,22,36,40,41] investigated the effect of saffron on HDL and LDL. Three of them presented non-significant changes in improving LDL levels in the adult population receiving saffron, while one found that saffron significantly reduced LDL cholesterol levels, with significant heterogeneity [41]. A significantly positive effect of saffron on HDL cholesterol levels was also reported by Tahmasbi et al. in their meta-analysis that showed a significant increase in HDL levels after saffron administration in the subgroup of obese/overweight adults with CAD [21] and also by Asbaghi et al., who demonstrated an overall increase in HDL levels [22]. Finally, triglyceride levels present a significant improvement after saffron supplementation in two metanalyses in obese adults [22,37]. However, this finding is not consistent, since triglyceride levels remain unchanged after saffron intervention in other obese/overweight adult populations pooled in various meta-analyses [21]. High heterogeneity among studies is the main source of discrepancy among the different results. It is of major importance to have access to high-quality, homogenous data in order to confidently draw conclusions.

The strength of the present study is mainly based on the selection of the study population. Young adolescents with obesity and prediabetes are a patient group with very few therapeutic options for the clinician. New pharmacological agents like GPL-1 agonists have recently been approved for adolescent obesity, but these options are very limited, significantly expensive, and moderately tolerated as they are only injectable [42]. The long-term cost-effectiveness of pharmacological therapeutic options is also highly controversial or even unfavorable, compared to simple lifestyle modification [43].

Thus, focusing on potential nutraceutical agents for use during adolescence is of major importance. For this reason, the present study tried to develop the known effects of nutraceutical saffron in adolescents, for the first time, in order to provide evidence to enhance therapeutics options in adolescent obesity and prediabetes. Additionally, the hereby studied nutraceutical agent, Saffron Kozanis, is a species of saffron with sparse data available so far. Finally, the blinding of the study design, which was randomized-controlled, provides a certain background to the extracted data. Despite providing new evidence, the result of the present study should be evaluated with the recognition of certain limitations. The intervention included a fixed dosage given to all participants in each study arm. Possible adjustment of the intervention dosage on participants’ anthropometry would provide more accurate conclusions and a dosage- dependent effect. In addition, dietary factors that may act as confounders were not assessed in this study design. Finally, the protocol evaluated the whole stigma of the medicinal plant (Saffron Kozanis) as an anti-obesity agent; therefore, we could not clarify with certainty which of the biological components of saffron possess its positive effects.

## 5. Conclusions

The current double-blind, randomized, placebo-controlled study using the medicinal plant named Saffron Kozanis for the first time in children and adolescents with obesity demonstrates its remarkable effects in successful weight management and improvement of lipid profiles. However, glucose metabolism was not found to be affected. Clearly, larger patient cohorts are needed to confirm these findings. The possibility of using products from nature instead of traditional medicines for health problems is a great prospect and challenge both for the compliance and quality of life of young people and for the economy of societies.

## Figures and Tables

**Table 1:** Baseline characteristics of adolescent participants in the study groups.

Variable	Study Groups			*p*-Value
	Crocus Sativus (*n* = 25)	Metformin (*n* = 25)	Placebo (*n* = 24)	
Gender (male/female)	12/13	11/13	11/13	0.841
Age (years)	12.32 ± 1.43	12.59 ± 1.44	12.27 ± 1.87	0.626
Height (cm)	159.60 ± 10.94	159.73 ± 10.04	156.93 ± 14.97	0.386
Height z-score	1.00 ± 1.37	0.89 ± 0.80	0.86 ± 1.72	0.802
Weight (kg)	73.43 ± 15.28	76.31 ± 12.27	74.71 ± 24.7	0.431
Weight z-score	2.16 ± 0.49	2.23 ± 0.43	2.18 ± 0.73	0.545
BMI	28.62 ± 3.47	29.78 ± 3.39	29.72 ± 5.83	0.422
BMI z-score	2.04 ± 0.31	2.07 ± 0.30	2.07 ± 0.38	0.802
WC (cm)	95.27 ± 9.07	96.38 ± 7.12	95.05 ± 4.13	0.561
SBP (mmHg)	116.21 ± 9.73	119.44 ± 10.46	112.96 ± 10.43	0.128
DBP (mmHg)	69.00 ± 10.81	73.72 ± 9.51	72.58 ± 9.42	0.237

Numerical variables are expressed as mean ± SD or median (range) and categorical variables are expressed as number (%). *p*-value: between group comparisons from chi-square test for qualitative variables, and one-way ANOVA or Kruskal–Wallis test for quantitative variables, as appropriate. BMI: body mass index, WC: waist circumference, SBP: systolic blood pressure, DBP: diastolic blood pressure

**Table 2:** Comparison of anthropometric and clinical indices among study groups.

Variable		Study Groups			*p*-Value *
		Crocus Sativus (*n* = 25)	Metformin (*n* = 25)	Placebo (*n* = 24)	
Height (cm)	Before	159.60 ± 10.94	159.73 ± 10.04	156.93 ± 14.97	
	After	161.07 ± 11.04	161.00 ± 10.40	158.54 ± 14.62	0.746
	*p*-value **	<0.001	<0.001	<0.001	
Height z-score	Before	1.00 ± 1.37	0.89 ± 0.80	0.86 ± 1.72	
	After	1.00 ± 1.37	0.89 ± 0.86	0.89 ± 1.73	0.951
	*p*-value **	0.802	0.967	0.131	
Weight (kg)	Before	73.43 ± 15.28	76.31 ± 12.27	74.71 ± 24.7	
	After	72.49 ± 15.59	72.12 ± 12.89	76.02 ± 23.78	0.374
	*p*-value **	0.092	<0.001	0.008	
Weight z-score	Before	2.16 ± 0.49	2.23 ± 0.43	2.18 ± 0.73	
	After	2.02 ± 0.56	1.93 ± 0.55	2.24 ± 0.76	0.224
	*p*-value **	<0.001	<0.001	0.269	
BMI	Before	28.62 ± 3.47	29.78 ± 3.39	29.72 ± 5.83	
	After	27.71 ± 3.55	27.64 ± 3.26	29.68 ± 5.43	0.076
	*p*-value **	<0.001	<0.001	0.781	
BMI z-score	Before	2.04 ± 0.31	2.07 ± 0.30	2.07 ± 0.38	
	After	1.90 ± 0.33	1.81 ± 0.35	2.06 ± 0.37	0.052
	*p*-value **	<0.001	<0.001	0.496	
WC (cm)	Before	95.27 ± 9.07	96.38 ± 7.12	95.05 ± 4.13	
	After	92.75 ± 9.27	92.36 ± 8.15	95.29 ± 13.45	0.499
	*p*-value **	0.042	<0.001	0.710	
SBP (mmHg)	Before	116.21 ± 9.73	119.44 ± 10.46	112.96 ± 10.43	
	After	115.04 ± 8.77	117.52 ± 8.83	113.91 ± 10.71	0.605
	*p*-value **	0.553	0.416	0.729	
DBM (mmHg)	Before	69.00 ± 10.81	73.72 ± 9.51	72.58 ± 9.42	
	After	69.58 ± 7.59	68.28 ± 7.23	70.62 ± 8.36	0.398
	*p*-value **	0.812	0.037	0.454	

Numerical variables are expressed as mean ± SD. *p*-value * between group comparison for post-intervention group differences based on ANCOVA adjusted for gender and age. *p*-value ** within group comparison based on paired sample *t*-test or Wilcoxon signed-rank test, as appropriate. BMI: body mass index, WC: waist circumference, SBP: systolic blood pressure, DBP: diastolic blood pressure

**Table 3:** Mean of differences between anthropometric and clinical indices among study groups before and post-intervention.

Variable	Group	Dif Mean ± SD	*p*-Value *	*p*-Value **	*p*-Value ***
Weight (kg)	Crocus sativus	−1.02 ± 2.91			
	Metformin	−4.18 ± 4.50	<0.001	0.006	0.001
	Placebo	+1.30 ± 2.23			
Weight z-score	Crocus sativus	−0.14 ± 0.14			
	Metformin	−0.39 ± 0.22	<0.001	0.010	<0.001
	Placebo	−0.05 ± 0.26			
BMI	Crocus sativus	−0.95 ± 1.11			
	Metformin	−2.15 ± 1.91	<0.001	0.013	0.003
	Placebo	−0.04 ± 0.72			
BMI z-score	Crocus sativus	−0.14 ± 0.13			
	Metformin	−0.26 ± 0.18	<0.001	0.016	<0.001
	Placebo	−0.01 ± 0.08			
WC (cm)	Crocus sativus	−2.66 ± 6.19			
	Metformin	−4.01 ± 4.88	0.004	0.579	0.027
	Placebo	+0.24 ± 3.19			
SBP (mmHg)	Crocus sativus	−1.12 ± 9.30			
	Metformin	−1.92 ± 11.60	0.856	0.683	0.779
	Placebo	+0.95 ± 13.37			
DBM (mmHg)	Crocus sativus	−4.23 ± 11.61			
	Metformin	−5.44 ± 12.33	0.260	0.109	0.496
	Placebo	−1.95 ± 12.59			

Numerical variables are expressed as mean ± SD. *p*-value * for one-way ANOVA or Kruskal–Wallis test for quantitative variables, as appropriate among the three groups. *p*-value ** for Student’s *t*-test or Mann–Whitney U test for as appropriate for comparison between Crocus sativus and metformin groups. *p*-value *** for Student’s *t*-test or Mann–Whitney U test for as appropriate for comparison between Crocus sativus and placebo groups. BMI: body mass index, WC: waist circumference, SBP: systolic blood pressure, DBP: diastolic blood pressure

**Table 4:** Comparison of glycemic indices among study groups.

Variable		Study Groups			*p*-Value *
		Crocus Sativus (*n* = 25)	Metformin (*n* = 25)	Placebo (*n* = 24)	
Fasting Glucose (mg/dL)	Before	103.04 ± 9.28	103.80 ± 12.41	103.58 ± 6.16	0.966
	After	99.96 ± 9.48	95.80 ± 12.16	98.66 ± 10.61	0.471
	*p*-value **	0.218	0.036	0.083	
Fasting Insulin (μIU/mL)	Before	22.84 ± 8.78	22.91 ± 6.51	22.30 ± 7.24	0.955
	After	19.96 ± 10.59	17.18 ± 7.83	23.35 ± 9.05	0.065
	*p*-value **	0.192	0.004	0.440	
HbA1c%	Before	5.33 ± 0.28	5.30 ± 0.19	5.28 ± 0.26	0.738
	After	5.25 ± 0.33	5.27 ± 0.19	5.19 ± 0.28	0.496
	*p*-value **	0.183	0.513	0.100	
HOMA-IR	Before	5.83 ± 2.41	5.91 ± 2.02	5.71 ± 1.92	0.951
	After	5.02 ± 2.85	4.07 ± 1.91	5.83 ± 2.92	0.063
	*p*-value **	0.184	0.001	0.804	
Fasting Cholesterol (mg/dL)	Before	157.08 ± 23.85	154.04 ± 26.97	150.46 ± 20.80	0.633
	After	154.04 ± 22.78	142.60 ± 33.40	148.37 ± 18.96	0.281
	*p*-value **	0.415	0.003	0.288	
Fasting HDL (mg/dL)	Before	45.62 ± 9.52	45.84 ± 13.45	46.79 ± 8.22	0.931
	After	48.29 ± 8.37	47.76 ± 11.87	46.66 ± 10.99	0.820
	*p*-value **	0.023	0.103	0.906	
Fasting LDL (mg/dL)	Before	86.44 ± 21.56	84.20 ± 21.52	82.58 ± 18.40	0.774
	After	86.72 ± 20.02	74.28 ± 27.20	80.91 ± 16.95	0.116
	*p*-value **	0.931	0.001	0.271	
Fasting Triglycerides (mg/dl)	Before	100.08 ± 36.65	98.24 ± 43.41	97.12 ± 11.43	0.933
	After	81.20 ± 33.71	96.08 ± 50.5	101.25 ± 24.70	0.178
	*p*-value **	<0.001	0.827	0.293	
Uric Acid (mg/dL)	Before	4.86 ± 1.08	5.13 ± 1.07	5.03 ± 1.11	0.545
	After	4.96 ± 1.04	4.94 ± 1.10	5.15 ± 0.95	0.722
	*p*-value **	0.368	0.067	0.341	
Urea (mg/dL)	Before	23.12 ± 7.29	25.60 ± 7.39	25.95 ± 4.89	0.222
	After	25.52 ± 5.80	24.12 ± 5.08	24.82 ± 5.82	0.864
	*p*-value **	0.066	0.274	0.417	
Creatinine (mg/dL)	Before	0.56 ± 0.09	0.59 ± 0.11	0.56 ± 0.11	0.504
	After	0.59 ± 0.13	0.61 ± 0.10	0.55 ± 0.11	0.276
	*p*-value **	0.100	0.369	0.451	

Numerical variables are expressed as mean ± SD. *p*-value * among group comparison for group differences based on ANCOVA adjusted for gender and age. *p*-value ** within group comparison based on paired sample *t*-test. HOMA-IR: Homeostatic model assessment of insulin resistance, HDL: high-density lipoprotein cholesterol, LDL: low-density lipoprotein cholesterol.

**Table 5:** Mean of differences between glycemic indices among study groups before and post-intervention.

Variable	Group	Dif Mean ± SD	*p*-Value *	*p*-Value **	*p*-Value ***
Fasting Glucose (mg/dL)	Crocus sativus	−3.08 ± 12.17			
	Metformin	−8.00 ± 18.05	0.159	0.082	0.307
	Placebo	−4.92 ± 13.28			
Fasting Insulin (μIU/mL)	Crocus sativus	−2.87 ± 10.72			
	Metformin	−5.74 ± 8.96	0.005	0.256	0.039
	Placebo	+1.05 ± 6.55			
HbA1c%	Crocus sativus	−0.08 ± 0.23			
	Metformin	−0.02 ± 0.18	0.643	0.330	0.791
	Placebo	−0.08 ± 0.23			
HOMA-IR	Crocus sativus	−0.80 ± 2.95			
	Metformin	−1.84 ± 2.33	0.024	0.233	0.215
	Placebo	+0.13 ± 2.50			
Fasting Cholesterol (mg/dL)	Crocus sativus	−2.92 ± 17.57			
	Metformin	−11.44 ± 17.44	0.057	0.054	0.347
	Placebo	+2.08 ± 9.38			
Fasting HDL (mg/dL)	Crocus sativus	+2.56 ± 5.26			
	Metformin	+1.92 ± 5.67	0.013	0.598	0.008
	Placebo	−0.13 ± 5.12			
Fasting LDL (mg/dL)	Crocus sativus	+0.28 ± 15.92			
	Metformin	−9.92 ± 12.35	0.013	0.012	0.616
	Placebo	−1.66 ± 7.23			
Fasting Triglycerides (mg/dL)	Crocus sativus	−18.88 ± 22.32			
	Metformin	−2.16 ± 48.84	0.004	0.086	<0.001
	Placebo	+4.12 ± 18.78			
Uric acid (mg/dL)	Crocus sativus	−0.08 ± 0.44			
	Metformin	−0.19 ± 0.49	0.141	0.117	0.763
	Placebo	+0.12 ± 0.60			
Urea (mg/dL)	Crocus sativus	+2.12 ± 5.39			
	Metformin	−1.48 ± 6.60	0.158	0.082	0.120
	Placebo	−1.08 ± 6.41			
Creatinine (mg/dL)	Crocus sativus	−0.02 ± 0.07			
	Metformin	−0.01 ± 0.06	0.150	0.695	0.075
	Placebo	−0.01 ± 0.05			

Numerical variables are expressed as mean ± SD. *p*-value * for one-way ANOVA or Kruskal–Wallis test for quantitative variables, as appropriate among the three groups. *p*-value ** for Student’s *t*-test or Mann–Whitney U test for as appropriate for comparison between Crocus sativus and metformin groups. *p*-value *** for Student’s *t*-test or Mann–Whitney U test for as appropriate for comparison between Crocus sativus and placebo groups. HOMA-IR: homeostatic model assessment of insulin resistance, HDL: high-density lipoprotein cholesterol, LDL: low-density lipoprotein cholesterol.

## Data Availability

Data is contained within the article.
